# Epigenetic Inheritance across the Landscape

**DOI:** 10.3389/fgene.2016.00189

**Published:** 2016-10-25

**Authors:** Amy V. Whipple, Liza M. Holeski

**Affiliations:** Department of Biological Sciences and Merriam-Powell Center for Environmental Research, Northern Arizona UniversityFlagstaff, AZ, USA

**Keywords:** epigenetics, transgenerational plasticity, landscape genomics, adaptation, epigenome, phenotype

## Abstract

The study of epigenomic variation at the landscape-level in plants may add important insight to studies of adaptive variation. A major goal of landscape genomic studies is to identify genomic regions contributing to adaptive variation across the landscape. Heritable variation in epigenetic marks, resulting in transgenerational plasticity, can influence fitness-related traits. Epigenetic marks are influenced by the genome, the environment, and their interaction, and can be inherited independently of the genome. Thus, epigenomic variation likely influences the heritability of many adaptive traits, but the extent of this influence remains largely unknown. Here, we summarize the relevance of epigenetic inheritance to ecological and evolutionary processes, and review the literature on landscape-level patterns of epigenetic variation. Landscape-level patterns of epigenomic variation in plants generally show greater levels of isolation by distance and isolation by environment then is found for the genome, but the causes of these patterns are not yet clear. Linkage between the environment and epigenomic variation has been clearly shown within a single generation, but demonstrating transgenerational inheritance requires more complex breeding and/or experimental designs. Transgenerational epigenetic variation may alter the interpretation of landscape genomic studies that rely upon phenotypic analyses, but should have less influence on landscape genomic approaches that rely upon outlier analyses or genome–environment associations. We suggest that multi-generation common garden experiments conducted across multiple environments will allow researchers to understand which parts of the epigenome are inherited, as well as to parse out the relative contribution of heritable epigenetic variation to the phenotype.

## Introduction

Understanding the ecological and evolutionary processes governing landscape patterns of genetic diversity and adaptive variation is important to predicting and managing the impacts of climate change on plant species distributions and function (Sork et al., 2013). Genomic, phenotypic, and environmental data are used to disentangle genetic and environmental influences on the phenotype and understand the distribution of adaptive variation among natural populations (Barrett and Hoekstra, 2011; Korte and Farlow, 2013; Lepais and Bacles, 2014; Rellstab et al., 2015). Evidence of adaptive differences across environmental gradients is common, but not universal (Sexton et al., 2014). There is increasing recognition that the inclusion of epigenetic-based transgenerational plasticity is likely to improve our understanding of adaptive phenotypic variation across the landscape (e.g., Cushman, 2014; Verhoeven et al., 2016). Here, we summarize the relevance of epigenetic inheritance to ecological and evolutionary processes, and review the literature on landscape-level patterns of epigenetic variation (both transgenerational and not). Then, we discuss the implications of transgenerational epigenetic variation for various approaches to landscape genomics. Finally, we discuss designs that can partition the landscape distribution of adaptive genetic and epigenetic variation.

Epigenetic modifications are changes in phenotype that are mediated by the regulation of gene expression rather than alterations in the DNA sequence. Epigenetic modifications may be reset within an organism’s lifespan or during meiosis, or they may be passed to offspring (Richards, 2006). These modifications can be inherited both maternally and paternally, via mechanisms such as DNA methylation, histone modification, and RNAi (Rapp and Wendel, 2005). The evolutionary relevance of transgenerational plasticity rests upon whether responses are adaptive and whether there is heritable genetic or epigenetic variation for the epigenetic modification (Day and Bonduriansky, 2011; Herman et al., 2014).

### Approaches in Landscape-level Investigations of Genomic Variation

Landscape-level investigations of patterns of genomic variation in plants have examined both adaptive and neutral loci and have employed a wide variety of methods. Genomic regions influencing adaptive traits are routinely discovered via genome–phenotype associations, phenotype-free approaches, and common gardens (Sork et al., 2013; Rellstab et al., 2015). Genome-wide association studies (GWAS) identify genotype–phenotype associations and can be done using plants grown in common gardens or from natural populations (e.g., Ingvarsson and Street, 2011). Phenotype-free approaches use genomic information to detect signatures of selection. Examples of these include outlier and environmental association analyses (EAA) (Rellstab et al., 2015). Outlier analyses detect loci that show evidence of strong selection, relative to the bulk of assayed loci that show effects of only population structure and drift. EAA refers to a number of statistical methods for detecting association between environmental variables and particular loci (Rellstab et al., 2015). Finally, growing multiple genotypes within the same common garden environment allows the genetic basis of a phenotype to be identified. When the same genotype is grown in multiple common gardens, the approach can also be used to examine the effect of environment on phenotype (Clausen et al., 1948).

### Landscape Genomic Patterns and Missing Heritability in Plants

Examination of gene flow within a species, based on neutral markers, typically shows isolation by distance (IBD) and/or isolation by environment (IBE). Genetic drift is the primary driver of IBD, where genetic differentiation increases with spatial distance (Wright, 1943; Charlesworth et al., 2003). IBE is influenced more by selection than is IBD, with genetic differentiation increasing with environmental distance (Bradburd et al., 2013; Wang and Bradburd, 2014). A review by Sexton et al. (2014) found both IBD and IBE were drivers of molecular genetic variation in natural plant populations, with IBD being more common than IBE.

Experiments combining an assessment of adaptive traits, molecular genetic loci underlying traits, genetic correlations, and gene flow barriers (distance, timing, and selection against immigrants) have thus far provided the most mechanistic understanding of landscape-level patterns of genetic variation (Lepais and Bacles, 2014). Often the realized heritability of populations detected in common garden and/or quantitative genetic designs cannot be fully explained by the loci detected in genomic approaches. The missing heritability problem can potentially be explained by a failure to detect loci of small effect or epistatic interactions among loci, but inherited (transgenerational) epigenetic variation is likely to be another source of the so-called missing heritability (Goldstein, 2009; Furrow et al., 2011). Epigenetic variation thus has potential implications for landscape-level adaptation.

### Ecological and Evolutionary Relevance of Epigenetic Inheritance

Initial population-level work studying epigenetic inheritance has demonstrated the substantial impacts of epigenetic factors on phenotypic variation in traits such as floral symmetry and defense against herbivores and pathogens (Cubas et al., 1999; reviews in Kalisz and Purugganan, 2004; Herman and Sultan, 2011; Holeski et al., 2012). Most investigations of the adaptive role of epigenetic modification have focused on DNA methylation patterns (Cervera et al., 2002; Verhoeven et al., 2016). Use of epiRILs, recombinant inbred lines that differ primarily in epigenetic status, in *Arabidopsis thaliana*, revealed epigenetic quantitative trait loci that account for 60–90% of the heritability in two ecologically relevant traits, flowering time and primary root length (Cortijo et al., 2014). These lines of research have led to the suggestion that heritable epigenetic variation could be the source of “missing heritability” not identified by QTL and GWAS studies (Bonduriansky, 2012).

Epigenetic-based transgenerational inheritance is predicted to have particular relevance for evolution in scenarios in which genetic variation alone may not provide sufficient trait variation to result in a robust response to selection (Jablonka and Raz, 2009). These scenarios might include: rapidly changing environments, such as those predicted by climate change models; species with low genetic variation due to asexual reproduction or founder effects; and organisms with long generation times (Bossdorf et al., 2008; Bonduriansky and Day, 2009; Nicotra et al., 2010; Castonguay and Angers, 2012). Despite potentially greater importance in the evolution of long-lived and asexual species, most empirical work so far has been done in sexually reproducing annuals (but see Richards et al., 2012; Zas et al., 2013; Yakovlev et al., 2014; Preite et al., 2015). Thus, not only may epigenetic-based transgenerational inheritance be a source of adaptive variation across a variety of species, it may be particularly important to organisms such as clonal grasses and long-lived trees, many of which are ecologically important foundation species.

## Epigenetic Patterns Across the Landscape

The potential adaptive significance of the epigenome suggests its relevance to studies of adaptation across the landscape. A number of very recent landscape-level studies have investigated the role of epigenetics in intra-specific trait variation and adaptation (Medrano et al., 2014; Dubin et al., 2015; Preite et al., 2015; Foust et al., 2016; Gugger et al., 2016; Herrera et al., 2016; Keller et al., 2016). These studies focus on at least one of the following: (i) the relationship between genetic and epigenetic variation at the landscape level, (ii) correlations between environmental variables and epigenetic status, and (iii) correlations between epigenetic status and plant functional traits.

Genetic and epigenetic variations are spatially structured across a landscape. A positive relationship between geographic distance and epigenetic differences across eight studies was identified by Herrera et al. (2016). This pattern is compatible with IBD patterns that are often found for genetic differences among populations, which are the main determinant of spatial genetic structure in plants (Sexton et al., 2014). Herrera et al. (2016) also found evidence in their case study that nearby individuals were more similar in their epigenome than in their genome, especially at small spatial scales. This suggests the potential for environmental influences on the epigenome, rather than a direct genome–epigenome relationship. Epigenomic patterns due to the environment may change through a lifespan, be regenerated each generation, or be inherited across generations. The results of several additional landscape-level surveys of epigenetic variation suggest that environmental factors are more important than spatial distance or the genome in shaping epigenetic structure (Schulz et al., 2014; Huang et al., 2015; Herrera et al., 2016). While this may suggest IBE in the genomic context (Sexton et al., 2014), the interpretation in the case of the epigenome is more complicated. Greater epigenomic than genomic differentiation suggests additional factors other than simple genomic determination are involved, such as adaptation via a heritable epigenome or direct effects of the environment on the epigenome.

Numerous studies have found correlations between epigenetic variation and environmental factors across a landscape (Dubin et al., 2015; Foust et al., 2016; Gugger et al., 2016; Keller et al., 2016). This supports the prediction that epigenetic-based transgenerational inheritance might be particularly relevant for evolution in rapidly changing environments, as well as the relevance of IBE to epigenetic diversity. Both genome-wide genetic and epigenetic variation in *Arabidopsis* were correlated with climate and spatial variables across Sweden and Eurasia (Keller et al., 2016). However, such correlations are not always found, as was the case for in dandelion (*Taraxacum officinale*) across a north–south transect from Luxembourg to central Sweden, where no gradient in DNA methylation was found (Preite et al., 2015). Correlations between epigenetic variation and the environment may be inconsistent between species in the same environments. In a study of five populations (including four overlapping sites), of two perennial salt marsh species (*Spartina alterniflora* and *Borrichia frutescens*), significant correlations were found between epigenetic variation and habitat in *S. alterniflora*, but not *B. frutescens* (Foust et al., 2016).

While a number of studies have investigated correlations between epigenetic patterns and the environment, far fewer have identified fitness-relevant phenotypes that are putatively altered via epigenetic mechanisms. We know of only two studies, both of the perennial herb *Helleborus foetidus*, that have investigated the influence of epigenetic variation on plant functional traits (Alonso et al., 2014; Medrano et al., 2014). One study was done at a landscape level, and found that 8% of functional trait variation was explained by methylation sensitive amplified polymorphisms. Multivariate functional trait diversity was correlated with epigenetic diversity after genetic diversity was taken into account. The authors suggest that epigenetic influence on functional traits allows *H. foetidus* to adapt to or survive in an array of environmental conditions (Medrano et al., 2014). The second study was done at a small-scale landscape level. Using plants from three sites within the same region in Spain, Alonso et al. (2014) found a negative correlation between global methylation and plant reproductive output.

Research to date demonstrates linkage between the environment and epigenomic variation within a generation, but demonstrating transgenerational inheritance requires more complex breeding and/or experimental designs. In many cases, regeneration of the epigenotype by environmental exposure in the current generation is a strong alternative interpretation. The development of labor-intensive tools such as epiRILs, from crosses between plants containing epigenetic changes induced by natural levels of biotic or abiotic stress are one mechanistic way to determine the heritability of stress-related epigenomic variation (Johannes et al., 2009; Holeski et al., 2012). Rearing individuals for generations in alternate environments would also give additional insights into the number of generations environmental signals persist in the epigenome.

## Landscape Genomics and the Epigenome

A critical next step is to determine the evolutionary relevance of the observed epigenetic patterns. Methods for detecting epigenetic variation at the landscape level are not designed to allow the researcher to differentiate between epigenetic variation that is reset within a generation and that which is inherited. In contrast to genetic or genomic patterns, the strength, and occurrence of transgenerational epigenetic inheritance at a landscape level, and thus its evolutionary implications, is poorly understood.

The evolutionary potential of transgenerational epigenetic variation is related to the degree to which it is inherited, as well as the extent to which it deviates from genetic variation. If genetic and epigenetic variations are strongly positively correlated, then the evolutionary trajectory of a population is not likely to deviate from that predicted by Mendelian patterns of inheritance (Day and Bonduriansky, 2011). In fact, in this case, the epigenome could be reset every generation and regenerated again from the genome with no influence of inheritance or current environment. In contrast, if genetic and epigenetic variation are weakly or not correlated, as has been demonstrated in *Arabidopsis* (Schmitz et al., 2013), then phenotypic change following selection could be decoupled from the genotype (Day and Bonduriansky, 2011; Liu, 2013). Evidence for both within-generation and transgenerational environmental influence on the epigenome (e.g., Saez-Laguna et al., 2014; Avramidou et al., 2015) suggests that complete correspondence between the genome and epigenome is unlikely. Thus, another expectation might be patterns of greater similarity of epigenomes in similar environments. This similarity could be further enhanced by inheritance of the epigenome.

Assessing the evolutionary relevance of epigenetic patterns across the landscape is a critical component in advancing the field of landscape-level studies of adaptation. How then, can this be done? Many of the methods currently used to detect adaptive genomic variation across the landscape (genome–phenotype associations, phenotype-free approaches, and/or common gardens) are not able to disentangle the effects of transgenerational epigenetic inheritance, relative to Mendelian genetic inheritance, on the phenotype.

Genome–phenotype association methods such as quantitative trait loci mapping and GWAS studies detect relationships between adaptive traits and genomic variation through the association of phenotypic and genomic data in individuals from crosses or natural populations (Rellstab et al., 2015). The inclusion of the phenotype in these analyses may complicate the interpretation of results because the influence of epigenetic effects on the phenotype remains unaccounted for. The results of phenotype-free approaches are relatively unaffected by the potential for transgenerational epigenetic inheritance because these analyses do not hinge on phenotypes that may integrate epigenetic influences. For example, outlier analyses use population genetic principles to detect loci that are likely to have experienced selection. Loci must be under sustained or strong selection to be detected, so this is a conservative technique that will miss many loci of small or fluctuating effect but will not be distorted by the occurrence of epigenetic variation. A second phenotype-free method, EAA, is based on genetic and environmental data and thus will also be unchanged by the occurrence of epigenetic variation. Environmental association analyses are being extended to analyze the association between the epigenome and the environment (Verhoeven et al., 2016), but the interpretation should include the possibilities of the genome and/or environment creating the epigenomic state without the involvement of transgenerational epigenetic inheritance.

## Critical Need for Common Garden Approaches

Common garden studies are crucial for disentangling environmental and genetic influences on adaptive traits. Common garden and quantitative genetic designs rarely cover a landscape in as much detail as methods such as GWAS. However, in combination with genomic data, these studies can be used to more fully understand patterns of adaptive variation across the landscape (Sork et al., 2013; De Kort et al., 2014; Lepais and Bacles, 2014).

Multi-generation common garden experiments conducted across multiple environments will allow researchers to understand adaptive epigenomic inheritance (Robertson and Richards, 2015). In plants, transgenerational effects not explained by differences in seed size or mass (the primary visible indications of offspring provisioning) and persisting for multiple generations are hypothesized to occur via epigenetic mechanisms (Zas et al., 2013). Demonstrating that neither genetic loci nor the environment of the individual is the sole source of epigenetic expression, and that epigenomic variation influences adaptive traits, would provide strong evidence for the evolutionary relevance of epigenetic inheritance.

**FIGURE 1:**
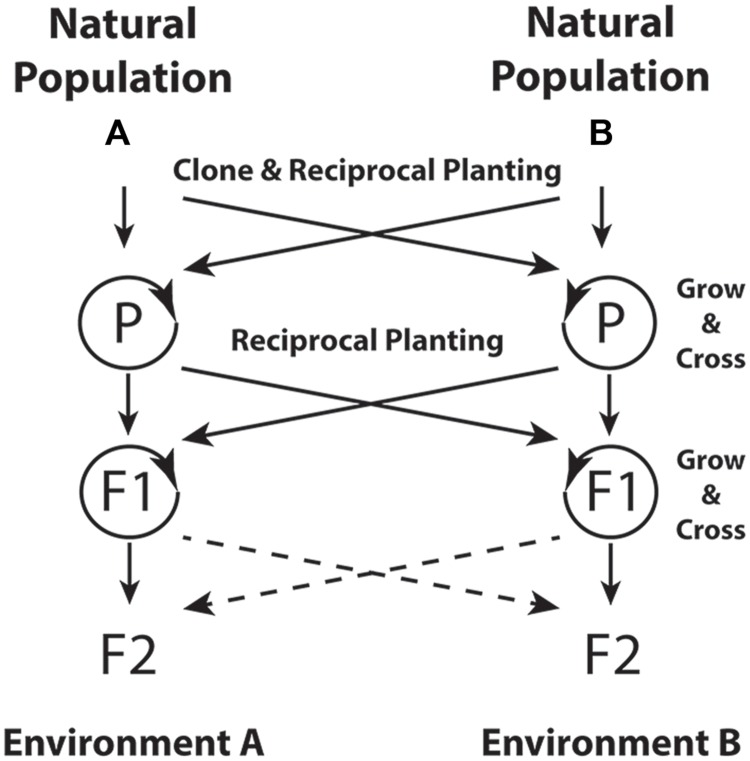
**Common garden design for distinguishing among genetic and epigenetic inheritance mechanisms for adaptive traits in a clonal plant species.** The reciprocal environmental exposure (Environments A and B) in the parental (P) generation is the primary treatment for the question of whether the environment induces heritable epigenetic changes. A comparison of trait values of clonal replicates or full siblings planted in Environment A and B and derived from parent(s) in either environment A or B will expose within-generation phenotypic plasticity. In the F_2_ generation, a comparison of trait values in offspring derived from the same parental genotypes grown in parental environments A versus B should expose phenotypic variation due to epigenetic inheritance. These offspring will share inherited genetic influences, and growth in the same environment eliminates the effects of within-generation plasticity. Additional generations or epigenomic assays may be needed to fully ascribe variation to epigenetic versus resource-based explanations. Dashed lines indicate optional reciprocal planting in the F_2_ generation to further understand the time scale and reversibility of environmentally induced inheritance effects.

Controlling for genetic sources among environments, and especially the use of clones, would give insight into the extent of genetic determination of the epigenome. Use of multiple environments and of plant sources that span a landscape will allow testing of adaptive hypotheses. **Figure 1** shows a design for distinguishing between genetic and non-genetic inheritance mechanisms for adaptive traits. In this design, natural populations with contrasting environments are used in a reciprocal transplant context to test the adaptive nature of variation. The use of a clonal plant species allows for greater control of genetic background across treatments, and clonal replicates can help researchers minimize the effects of somatic mutation during the experiment. Multiple generations and reciprocal crosses between environments and sources in the parental generation enable separation of the various inheritance patterns (i.e., Day and Bonduriansky, 2011). Assays of the epigenome or additional generations of testing would strengthen an inference of epigenetic inheritance as a contributing causal agent in adaptation to the environment. Inclusion of environments spanning a species range, and modeling to interpolate across the landscape, could create a bridge between traditional common garden and landscape genomic scales (Cushman, 2014).

Thus far, only a few studies have taken advantage of common garden approaches for studying epigenetic inheritance. In two examples, clonal systems also helped narrow down potential sources of phenotypic variation. Studies in *Pinus pinaster* (Cendán et al., 2013; Zas et al., 2013) used seed orchards with cloned genotypes in contrasting common garden environments to assay for effects of maternal environment on offspring traits. They demonstrated effects of maternal environment on offspring traits that could be explained by resources (seed mass) and additional effects that could not be attributed to seed mass. Wilschut et al. (2016) made use of an unusual landscape genetic structure in the dandelion (*T. officinale*) where the same clone is distributed across a wide geographic area. The clonal identity controlled for genetic variation (excluding mutation in these clonal lines since divergence). Generations in different environments resulted in epigenetic differentiation among locations. When plants were grown in a common environment, traits of clonal replicates from different environments remained distinct, showing differentiation was not caused by the environmental exposure in the current generation.

## Conclusion

Strong evidence exists for epigenetic inheritance and its potential to influence adaptive traits in plants. At the landscape level, studies have identified genomic variation that affects adaptation, but the genetic basis of additional phenotypic variation remains unaccounted for. A number of recent investigations of epigenetic variation across the landscape show patterns consistent with epigenetic inheritance contributing to adaptation. However, carefully designed common garden studies are needed to partition the contributions of genetic variation, phenotypic plasticity, and transgenerational epigenetic inheritance to adaptive phenotypes.

## Author Contributions

Both AW and LH contributed to the ideas, writing and editing of this manuscript.

## Conflict of Interest Statement

The authors declare that the research was conducted in the absence of any commercial or financial relationships that could be construed as a potential conflict of interest.
